# Supplementary material for: Cloud BioLinux: pre-configured and on-demand bioinformatics computing for the genomics community
Source: BMC Bioinformatics. 2012 Mar 19;13:42. doi: 10.1186/1471-2105-13-42 (PMC3372431; doi:10.1186/1471-2105-13-42)
Supplement: Additional file 1 — Supplementary 1 Cloud BioLinux software documentation in the form of a mini, self-contained website. Users need to download and uncompress the .zip file, and open through a web browser the "index.html" file available on the main directory. (ZIP 1823 kb). [file 1471-2105-13-42-S1.ZIP › Cloud-BioLinux-Package-Documentation/docs/gap4.html]

Bio-Linux Software Documentation Pages

Back to search form

## gap4

|  |  |
| --- | --- |
| Name | gap4 |
| Description | **Gap4** is a part of the Staden package of sequence assembly, editing and analysis tools.  **Gap4** performs assembly, contig joining, assembly checking, repeat searching, experiment suggestion, read pair analysis and contig editing. It has graphical views of contigs, templates, readings and traces which all scroll in register. Contig editor searches and experiment suggestion routines use phred confidence values to calculate the confidence of the consensus sequence and hence only identify places requiring visual trace inspection or extra data. The result is extremely rapid finishing and a consensus of known accuracy.  See also infomation about pregap4, spin and trev. |
| Homepage | http://sourceforge.net/projects/staden/ |
| Remote Documentation | http://staden.sourceforge.net/ |
